# Elevated levels of butyric acid in the jejunum of an animal model of broiler chickens: from early onset of *Clostridium perfringens* infection to clinical disease of necrotic enteritis

**DOI:** 10.1186/s40104-024-01105-5

**Published:** 2024-11-02

**Authors:** Hemlata Gautam, Noor Ahmad Shaik, Babajan Banaganapalli, Shelly Popowich, Iresha Subhasinghe, Lisanework E. Ayalew, Rupasri Mandal, David S. Wishart, Suresh Tikoo, Susantha Gomis

**Affiliations:** 1 Department of Veterinary Pathology, Western College of Veterinary Medicine, University of Saskatchewan, 52 Campus Drive, Saskatoon, S7N 5B4 Canada; 2 Department of Genetic Medicine, Faculty of Medicine, King Abdulaziz University, Jeddah, 21589 Saudi Arabia; 3 Department of Pathology and Microbiology, Atlantic Veterinary College, University of Prince Edward Island, 550 University Ave, Charlottetown, PE C1A 4P3 Canada; 4 Departments of Biological Sciences and Computing Science, University of Alberta, Edmonton, AB T6G 2E9 Canada; 5 Vaccinology and Immunotherapy, School of Public Health, University of Saskatchewan, Saskatoon, 7N 5E3 Canada

**Keywords:** Broiler chickens, Butyric acid, Gut health, Metabolic pathways, Necrotic enteritis, Toxin genes

## Abstract

**Background:**

Necrotic enteritis (NE) is an economically important disease of broiler chickens caused by *Clostridium perfringens* (CP). The pathogenesis, or disease process, of NE is still not clear. This study aimed to identify the alterations of metabolites and metabolic pathways associated with subclinical or clinical NE in CP infected birds and to investigate the possible variations in the metabolic profile of birds infected with different isolates of CP.

**Methodology:**

Using a well-established NE model, the protein content of feed was changed abruptly before exposing birds to CP isolates with different toxin genes combinations (*cpa*,* cpb2*,* netB*,* tpeL*; *cpa*, *cpb2*,* netB*; or *cpa*,* cpb2*). Metabolomics analysis of jejunal contents was performed by a targeted, fully quantitative LC-MS/MS based assay.

**Results:**

This study detected statistically significant differential expression of 34 metabolites including organic acids, amino acids, fatty acids, and biogenic amines, including elevation of butyric acid at onset of NE in broiler chickens. Subsequent analysis of broilers infected with CP isolates with different toxin gene combinations confirmed an elevation of butyric acid consistently among 21 differentially expressed metabolites including organic acids, amino acids, and biogenic amines, underscoring its potential role during the development of NE. Furthermore, protein-metabolite network analysis revealed significant alterations in butyric acid and arginine-proline metabolisms.

**Conclusion:**

This study indicates a significant metabolic difference between CP-infected and non-infected broiler chickens. Among all the metabolites, butyric acid increased significantly in CP-infected birds compared to non-infected healthy broilers. Logistic regression analysis revealed a positive association between butyric acid (coefficient: 1.23, *P* < 0.01) and CP infection, while showing a negative association with amino acid metabolism. These findings suggest that butyric acid could be a crucial metabolite linked to the occurrence of NE in broiler chickens and may serve as an early indicator of the disease at the farm level. Further metabolomic experiments using different NE animal models and field studies are needed to determine the specificity and to validate metabolites associated with NE, regardless of predisposing factors.

**Supplementary Information:**

The online version contains supplementary material available at 10.1186/s40104-024-01105-5.

## Background

Necrotic enteritis (NE) accounts for significant economic losses in the poultry industry. *Clostridium perfringens* (CP), is the causative agent of NE. It is Gram-positive, rod-shaped, anaerobic, and spore-forming bacteria that grows in various environments such as dust, soil, and litter. Reduction of in-feed prophylactic antimicrobials has led to an increased incidence of NE in recent years. CP is an opportunistic pathogen which induces NE by excessive bacterial proliferation and the production of toxins including CP alpha (CPA), CP beta (CPB), epsilon toxin (ETX), iota toxin (ITX), CP enterotoxin (CPE), necrotic enteritis B like toxin (NetB) and large clostridial toxin (TpeL) [1]. Several predisposing factors have been identified as factors that contribute to the adhesion and colonization of this bacteria in the intestinal mucosa. These factors include coccidia infections, immune suppressing viruses, high protein diets and abrupt changes in dietary protein. We have recently demonstrated an association of CP isolates with *netB* and *tpeL* toxin genes inducing severe pathological lesions of NE and associated dysbiosis of the intestinal microbiome in broiler chickens [2]. NE primarily affects broiler chickens 2–6 weeks of age, causing subclinical and clinical forms of NE. A sudden increase of mortality up to 50%, poor performance and disposal of carcasses at processing accounts for huge economic losses to the broiler industry worldwide [3]. According to the United States Center for Diseases Control and Prevention, NE is a primary health concern. In particular, CP was estimated to be the second most common cause of the foodborne illness due to consumption of contaminated poultry meat in the USA with about one million people affected by CP contaminated poultry products annually [4].

Outside of antibiotic treatment, there are no effective control strategies against NE and the management of the disease in broiler chickens. Host-pathogen interactions associated with the pathogenesis of NE, particularly changes of the intestinal microbiome at the onset of NE, are poorly understood [5]. An abrupt change in dietary protein along with CP challenge have been shown to decrease the abundance of *Lactobacillus* and *Oscillatoria* in the jejunum while increasing the abundance of *Clostridium* and *Escherichia* [2]. The cecum of CP infected birds had a compositional shift of *Lactobacillus* content where *L. johnsonnii*, *L. acidophilus* counts were significantly decreased and *L. reuteri* and *L. animalis* counts increased [6]. Dysbiosis of the gut not only alters gut microbiome composition but also gut-microbiota derived signaling molecules and metabolites. Gut microbial communities help in the transmission of hormone signals, the regulation of the host immune system and the production of key metabolites such as butyrate, propionate, and acetate [7]. A decrease of butyrate-producing strains in Ruminococcaceae and Lachnospiraceae families have been reported in a fishmeal and *Eimeria* co-infection model of NE in broiler chickens [6]. Upon colonization, pathogens have the capability to form biofilms and secrete toxic metabolites such as ammonia, phenol, para-cresol, and hydrogen sulfide amine, which further induces inflammation, DNA damage and intestinal permeability [7]. Lactic acid is the major microbial product of *Lactobacilli* via carbohydrate fermentation which further benefits lactate utilizing and butyrate producing bacteria [8]. CP infection has also been found to induce an inflammatory response in the jejunum and cecal tonsils by the release of interleukin (IL)-1β, IL-13 [T-helper 2 (Th2) cytokine], and IL-17 (Th17 cytokine) at day 7 post-challenge in broiler chickens [9].

Because metabolites are typically the downstream products of various upstream cellular regulatory processes, metabolites might act as potential biomarkers at the onset of NE [10]. Further exploration of these metabolites and metabolic pathways could assist in exploration of the NE disease process [11]. Host-pathogen interactions including the mechanisms by which intestinal microbial metabolic products regulate host metabolites are complex and dynamic [12]. Furthermore, production of metabolites during active infection further affects host-pathogen interactions. Pathogens are extremely malleable and can readily alter their gene expression to acquire nutrients from the host [13, 14]. CP lacks amino acid synthesis machinery; hence CP utilizes the resources from the host to produce enzymes such as sialidases, collagenases, hyaluronidases, and a variety of membrane damaging and pore-forming exo-toxins [1, 15]. All these activities are responsible for the severe diffuse necrosis seen in intestinal mucosa, which is characteristic of clinical NE in broiler chickens. In severe NE infections, the biosynthesis of tryptophan, tyrosine, phenylalanine (aromatic amines) and valine, leucine, isoleucine (branched amino acids) have been predicted (based on gene expression) to be increased in broiler chickens [16]. In birds with subclinical NE, indole, azelaic acid, oleic acid, valeric acid, 3-hydroxybenzoic acid, L-glutamic acid, hydro-cinnamic acid, pantothenic acid, nicotinic acid hypoxanthine and D-glucose were found to be upregulated compared to the healthy birds [17]. A number of compounds have been used to prevent intestinal cell death and inflammation associated with NE. For instance, the secondary bile acid, deoxycholic acid, has been shown to prevent NE-induced weight loss and to mitigate inflammatory cyclooxygenase (COX) responses [5]. Likewise, a mixture of medium-chain fatty acids and short-chain fatty acids (SCFAs) have been shown to reduce NE in broiler chickens [18].

This is the first study to our knowledge which focused on identifying subclinical and clinical NE associated metabolic markers in broiler chicken NE animal models developed without the use of *Eimeria* species. Therefore, our objectives were to (1) explore the jejunal metabolomic profile and metabolic pathways leading to subclinical and clinical NE in broiler chickens; and (2) study the possible impact of different toxin gene combinations carried by different CP isolates on the broiler chicken jejunal metabolomic profile and metabolic pathways leading to NE development.

## Methods

**Fig. 1:**
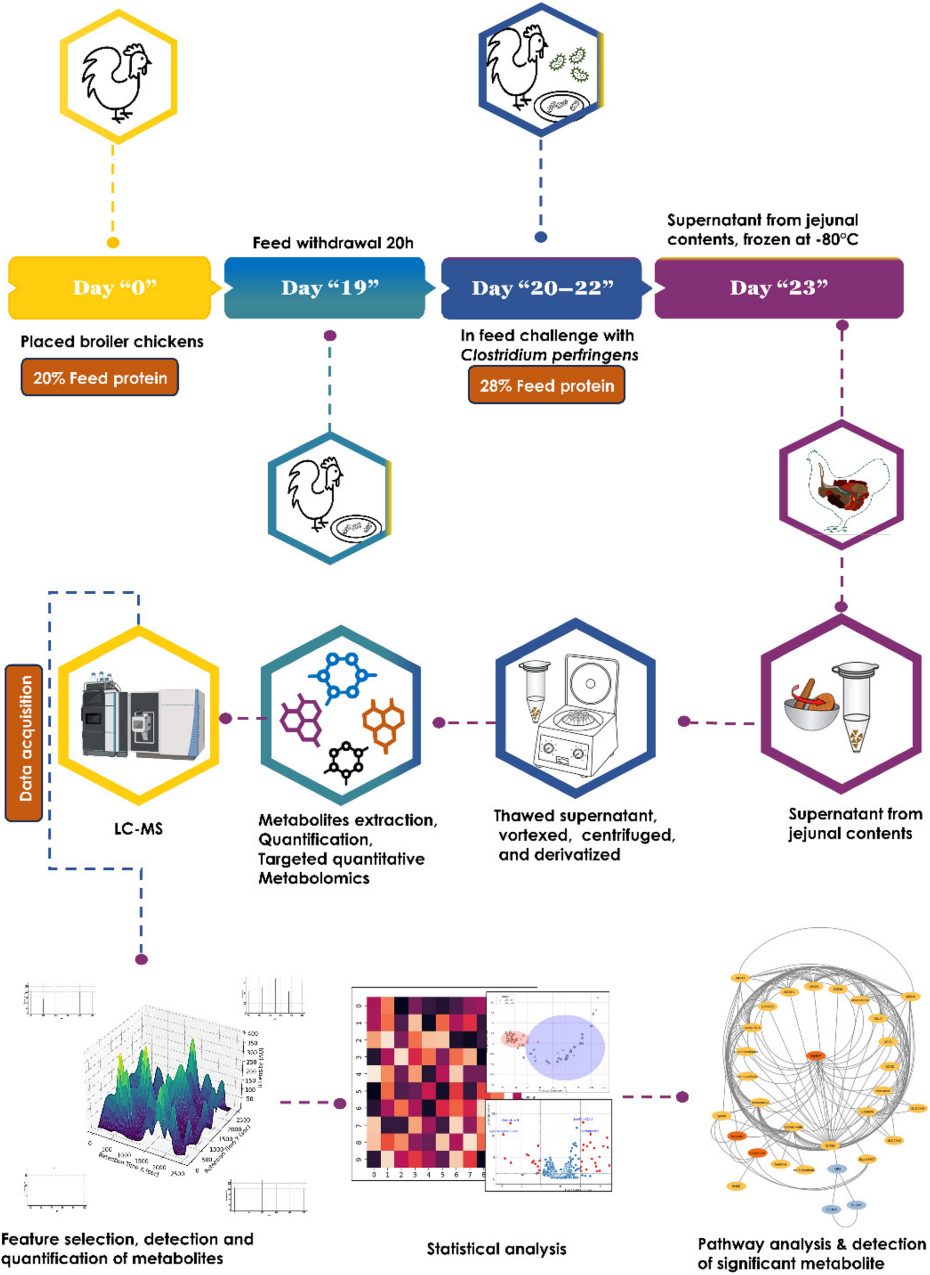
Integrated metabolomic workflow for evaluating the impact of CP challenge on broiler chicken metabolism. This flowchart outlines the timeline from chick placement to feed changes, pathogen challenge, and necropsy sampling. The workflow illustrates the metabolite extraction, LC-MS analysis, and subsequent data processing steps leading to the identification of significant metabolites associated with NE pathogenesis

The overall workflow for this study is presented in Fig. 1. All animal experiments conducted for this study were granted by the Animal Research Ethics Board at the University of Saskatchewan and Canadian Council on Animal Care guidelines followed. Animal work was conducted at the Animal Care Unit (ACU), Western College of Veterinary Medicine (WCVM), University of Saskatchewan. Birds were raised on soft wood shavings of depth 3–5 cm. From placement until 3 days of age, chicks received 23 h of light and 1 h of darkness at 40 lx. After 3 d, the darkness period increased to 8 h and the light intensity decreased to 30 lx during the 16 h light period. The initial temperature was set at 30–32 °C for first 3 d and decreased by 0.5 °C/d until a temperature of 21 °C was maintained. As described earlier [2], protein content of the feed was abruptly increased to induce NE post CP infection. Briefly, day-old broiler chickens were fed with a 20% protein raised without antibiotics (RWA) poultry starter (Farm Choice™ RWA, MasterFeeds, Canada) diet until 18 days of age. The 20% protein diet was then withdrawn at 19 days of age and replaced with 28% protein diet. The 28% protein diet was prepared by mixing 25% RWA turkey/game bird starter crumble (MasterFeeds, Canada) and 38% layer/grower supplement (MasterFeeds, Canada) at a ratio of 10:3. After 24 h, broilers were fed with 28% protein diet mixed with CP culture grown in Thioglycollate broth media in a ratio of 1:1 (v/w) for 3 d consecutively (20 to 22 days of age) (Table S1). Based on the severity of necrosis, macroscopic and microscopic lesions were scored as previously described [2].

### Experiment design

#### Metabolomics profile of the jejunum with subclinical NE, clinical NE and no NE following CP challenge

Day-old broiler chickens (*n* = 82) from a local hatchery in Saskatchewan, Canada (Prairie Pride Chick Sales, Grandora, SK, Canada) were placed at the ACU, WCVM, University of Saskatchewan, Canada. Birds were randomly allocated to 2 groups: (1) no CP challenge (*n* = 17) and (2) CP challenge with CP isolates containing *cpa*, *netB*, *cpb2* and *tpeL* genes [2] (*n* = 65). At 23 days of age, birds were euthanized and jejunal contents were collected from each animal. Based on gross and histopathological lesions of NE, jejunal contents were divided into 3 groups for metabolomics analysis: (a) subclinical NE, characterized by only histopathological lesions (*n* = 17); (b) clinical NE characterized by both gross and histopathological lesions of NE (*n* = 21); and (c) birds challenged with CP but no gross and histopathological lesions of NE (*n* = 27).

#### Metabolomics profile of the jejunum following challenge with different CP isolates

We explored the broiler chicken jejunal metabolic profile following challenge with different CP isolates carrying different combinations of toxin genes to understand the impact of CP toxins on the jejunal metabolome. The NE challenge experiment was conducted using the same NE infection model as described above. A total of 63 day-old broiler chicks (Prairie Pride Chick Sales, Grandora, SK, Canada) were randomly divided into four groups and challenged with CP as follows: (1) no CP challenge (*n* = 14); (2) CP isolate containing *cpa*, *netB*, *cpb2* and *tpeL* genes; (4T), (*n* = 17); (3) CP isolate containing *cpa*, *cpb2* and *netB* genes (3T), (*n* = 16); and (4) CP isolate containing *cpa* and *cpb2* genes (2T), (*n* = 16). Broilers were monitored for mortality and jejunal lesions scored at the end of the trial at 23 days of age as described above [2].

### Jejunal content collection

At 23 days of age, birds were euthanized and the entire length of the intestine was examined for gross lesions of NE. Histopathology samples were collected from sections of jejunum. A section of unopened jejunum was carefully removed and intestinal contents collected into sterile 5-mL Eppendorf tubes and placed on ice. Intestinal contents were then centrifuged for 5 min at 1,789 × *g* to separate intestinal debris. The supernatant was collected in Eppendorf tubes and flash frozen using dry ice and ethanol. The samples were stored at −80 °C until metabolomics analysis was performed at the Metabolomics Innovation Centre (TMIC), University of Alberta, AB, Canada.

### Metabolomic analysis

Reverse-phase LC-MS/MS and direct flow injection mass spectrometry (DFI-MS) was utilized to identify and quantify ≤ 150 targeted endogenous metabolites with the ABSciex 4000 QTrap mass spectrometer from Applied Biosystems/MDS Sciex. These metabolites include sugars, amino acids, acylcarnitines, uremic toxins, biogenic amines, glycerophospholipids, and sphingolipids [19]. The method detailed elsewhere [19], involved chemical derivatization (phenylisothiocyanate (PITC) and 3-nitrophenylhydrazine (3-NPH)) and extraction of analytes from the biofluid matrix, with mass-spectrometric detection and metabolite identification performed using multiple reaction monitoring (MRM) pairs. The ^13^C isotope-labeled internal standards were utilized for precise metabolite quantifications. The assay reactions were conducted in 96-well microplates, with blanks (*n* = 14), zero (*n* = 3), standards (*n* = 7) and quality control (*n* = 3) samples. Then the samples (except organic acids) ice thawed, vortexed, and centrifuged at 13,000 × *g*. Thereafter, 10 µL of sample was added into the microplate and then dried under a nitrogen stream. Sample derivatization was conducted by addition of PITC. Metabolite extraction was carried out by addition of 300 µL of extraction solvent. Then samples were centrifuged, diluted with MS running solvent for LC-MS injection.

The organic acids were analyzed by adding methanol (150 µL, ice-cold) and isotope-labeled internal standard (10 µL) mixture to samples (50 µL) and overnight incubation to precipitate proteins. The samples were then centrifuged at 13,000 × *g* for 20 min. The sample supernatant was collected and loaded on to wells in a microplate, and 3-NPH reagent added. Thereafter this mixture was allowed for 2-h incubation, followed by addition of butylated hydroxytoluene (BHT) stabilizer and water before LC-MS injection.

The mass spectrometry analysis was conducted using a tandem mass spectrometer (ABSciex 4000 Qtrap^®^, Applied Biosystems, CA, USA) equipped with an ultra-high performance liquid chromatography (UHPLC) system (Agilent 1260 series, Agilent Technologies, CA, USA). An LC method, followed by a direct injection (DI) method, was used to deliver samples to the mass spectrometer. Data acquisition, processing, and analysis steps were executed with the Analyst 1.6.2 software.

### Metabolomic data processing and visualization

All measured metabolite data underwent logarithmic transformation to normalize the data distribution. Missing values and values below the limit of detection (LOD) were replaced with zeros to ensure consistency and integrity in the dataset, thus minimizing potential biases in the analysis. Differential expression analysis of metabolites was conducted using the limma algorithm [20] focusing on comparisons such as combined (clinical + subclinical + challenged) vs. control, clinical vs. control, subclinical vs. control, and challenged vs. control.

### Statistical analysis

A univariate *t*-test analysis was used to assess individual metabolite differences between groups. *P*-values were adjusted for multiple comparisons using the False Discovery Rate (FDR) method, with a significant cutoff for adjusted *P*-values set at < 0.05. Box plots (ggpubr R package) were used to illustrate metabolite concentration differences across groups, while Volcano plots (Enhanced Volcano R package) highlighted differentially expressed metabolites, emphasizing those with log fold changes and *P*-values below the 0.05 significance threshold [22]. Venn diagrams (via an R package) and heatmaps (heatmap R package) were used to visualize overlapping metabolites and individual sample expression patterns, respectively [23]. Unsupervised principal component analysis (PCA) with ggplot2 was employed for data dimensionality reduction, identifying key metabolite patterns among groups [24, 25]. Logistic regression analysis (rms R package) explored relationships between metabolite levels and disease state [26]. For all statistical comparisons, *P* < 0.05 denotes statistical significance, *P* < 0.01 denotes strong significance, and *P* < 0.001 denotes extremely significance.

### Pathway analysis

The pathway analysis of the differentially expressed jejunal metabolites was performed using MetaboAnalyst version 5.0 [27], with *Gallus gallus* as the reference dataset. A *P* < 0.05 was considered statistically significant pathway. Pathway significance was depicted via a bubble plot, showing the ratio of detected metabolite hits to the total number of metabolites known to be in the pathway. Additionally, STITCH [28] analysis was conducted to explore metabolite-pathway interactions, with *Gallus gallus* selected as the organism of interest and a medium confidence level set at a C-score of 0.40. To visualize the resulting interaction networks, we utilized Cytoscape, a widely recognized software tool for network analysis and visualization [29].

## Results

### Jejunum metabolite content with subclinical NE, clinical NE and no NE following challenge

**Fig. 2:**
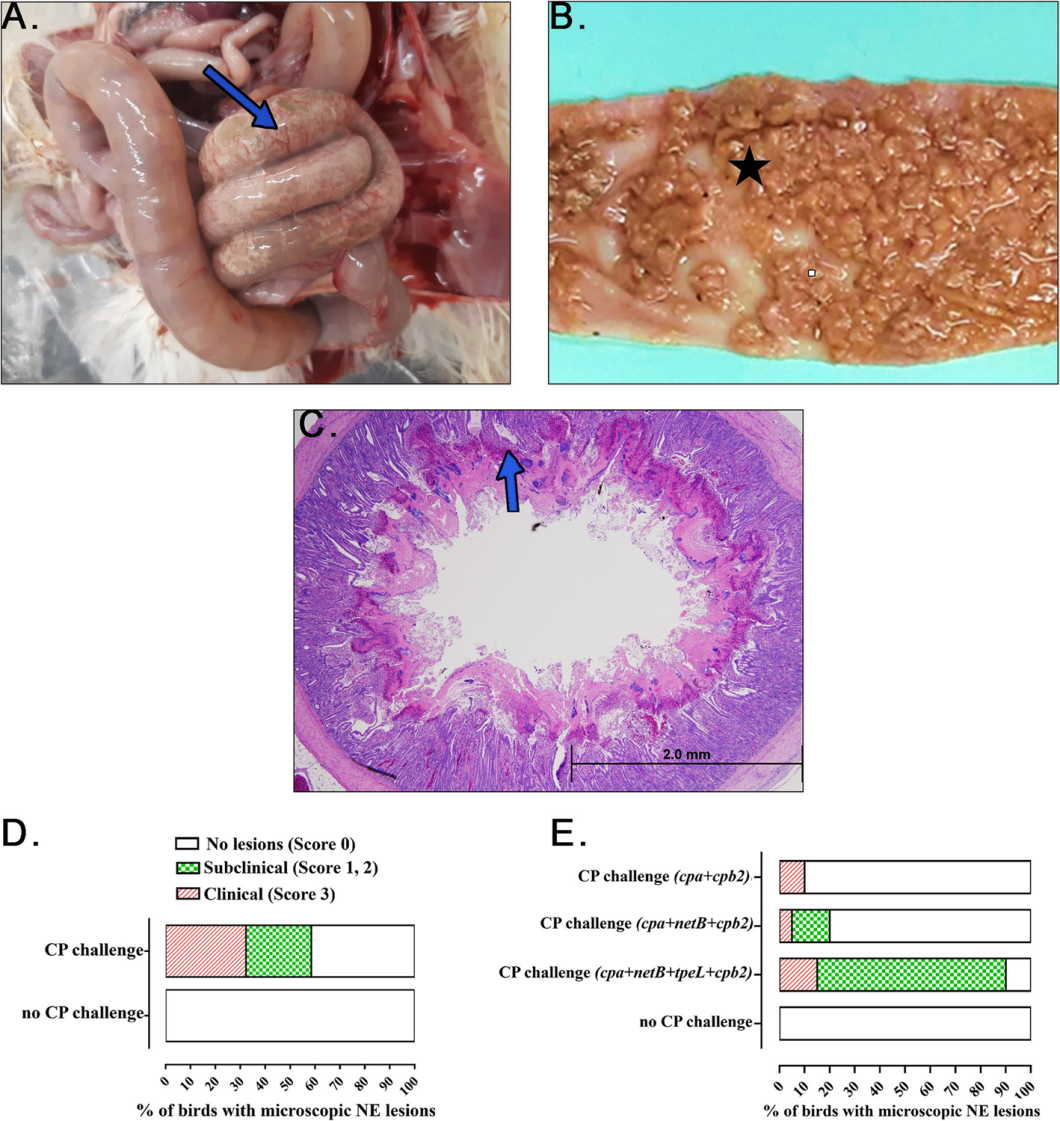
Gross and histopathological lesions of NE in broiler chickens. **A** Distended intestine with visible gas pockets and necrotic patches through the serosa, indicative of NE. **B** Opened jejunum mucosal surface with diffuse necrosis. **C** Histopathological evidence of diffuse coagulative necrosis of the intestinal villi. **D** The incidence of NE lesions in *Clostridium perfringens* (CP)-challenged versus negative control group, demonstrated a significant increase in lesion severity with CP challenge. **E** The bar graph shows the percentage of birds with microscopic NE lesions with different toxin gene combinations of CP challenge

Birds with NE had severe, diffuse necrosis of the intestine predominantly in the jejunum (Fig. 2A–C). Zero mortality was observed in groups 1 or 2 following CP challenge. No macroscopic (gross) or microscopic (histopathological) lesions of NE were detected in birds not challenged with CP (group 1). In contrast, birds challenged with CP had 58.5% of birds with NE lesions [score 1 and 2 in 26.2% (subclinical NE), score 3 in 32.3% (clinical NE) and remaining 41.5% birds had score 0 (no NE lesions following CP challenge)] (Fig. 2D).

**Fig. 3:**
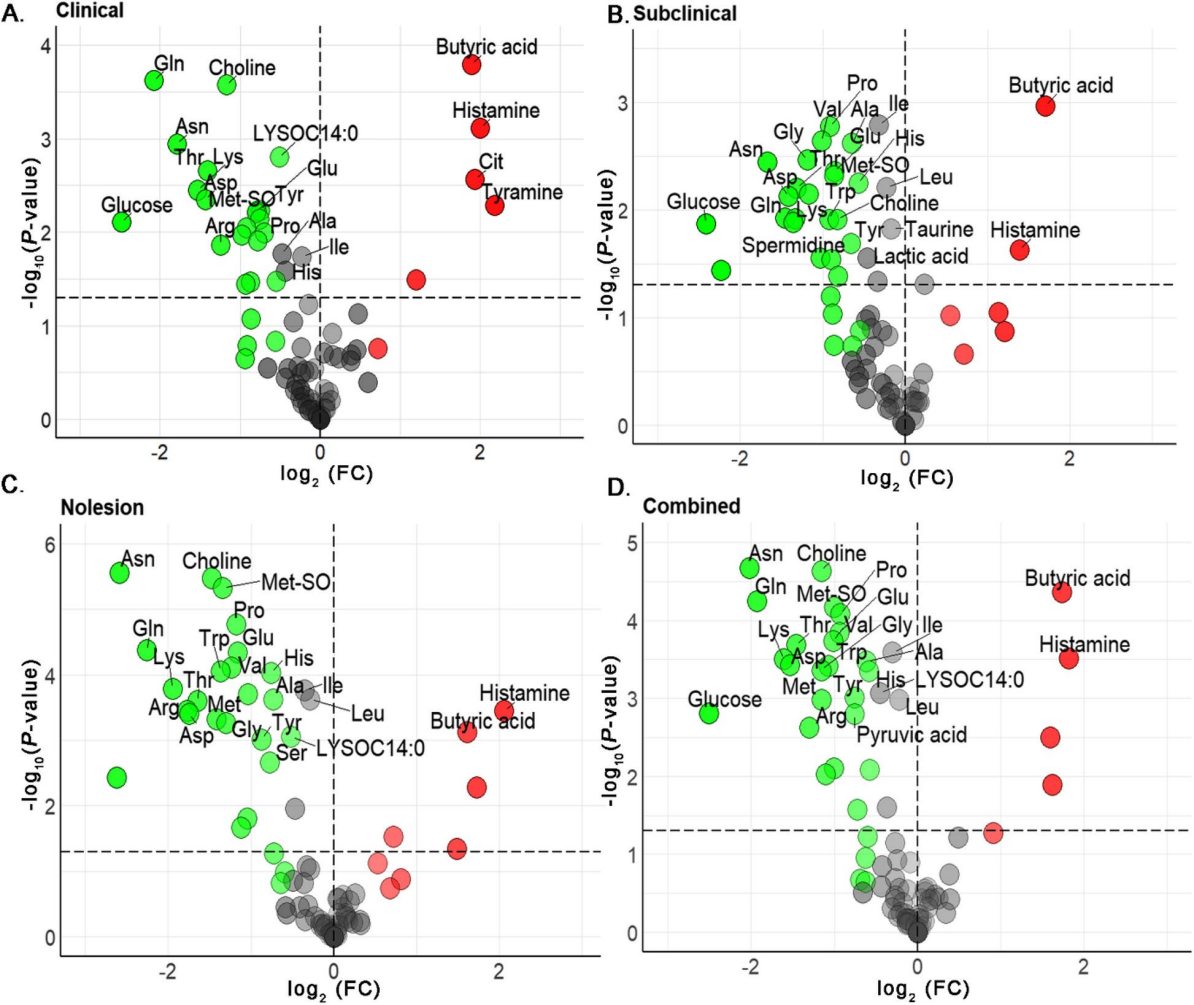
Metabolite expression in CP challenged broiler chickens. Volcano Plots (**A**–**D**) illustrate the statistical significance versus fold change of jejunal metabolites across clinical, subclinical, challenged-no lesions and combined NE groups compared to controls. Red dots represent significantly upregulated metabolites, and green dots indicate downregulated metabolites, showcasing distinct metabolic profiles associated with NE severity post CP challenge

 The absolute quantities of individual metabolites identified and quantified in jejunal contents of NE birds using reverse-phase LC-MS/MS and direct flow injection mass spectrometry (DFI-MS) are detailed in Table S2. The jejunal metabolomic analysis revealed distinct metabolic profiles across groups with clinical NE, subclinical NE, and no detectable NE. Volcano plots (Fig. 3A–D) distinguished these 3 groups, showing statistically significant metabolites as red dots. Compared to healthy controls, chickens with clinical NE had 9 upregulated and 37 downregulated metabolites, while birds with subclinical disease had 6 upregulated and 38 downregulated, while the birds that were challenged with CP, but which had no microscopic NE lesions, showed upregulation in 7 and downregulation in 47 metabolites. Overall, in all NE infected birds, there were 7 upregulated and 44 downregulated metabolites, with other comparisons to controls being non-significant.

**Fig. 4:**
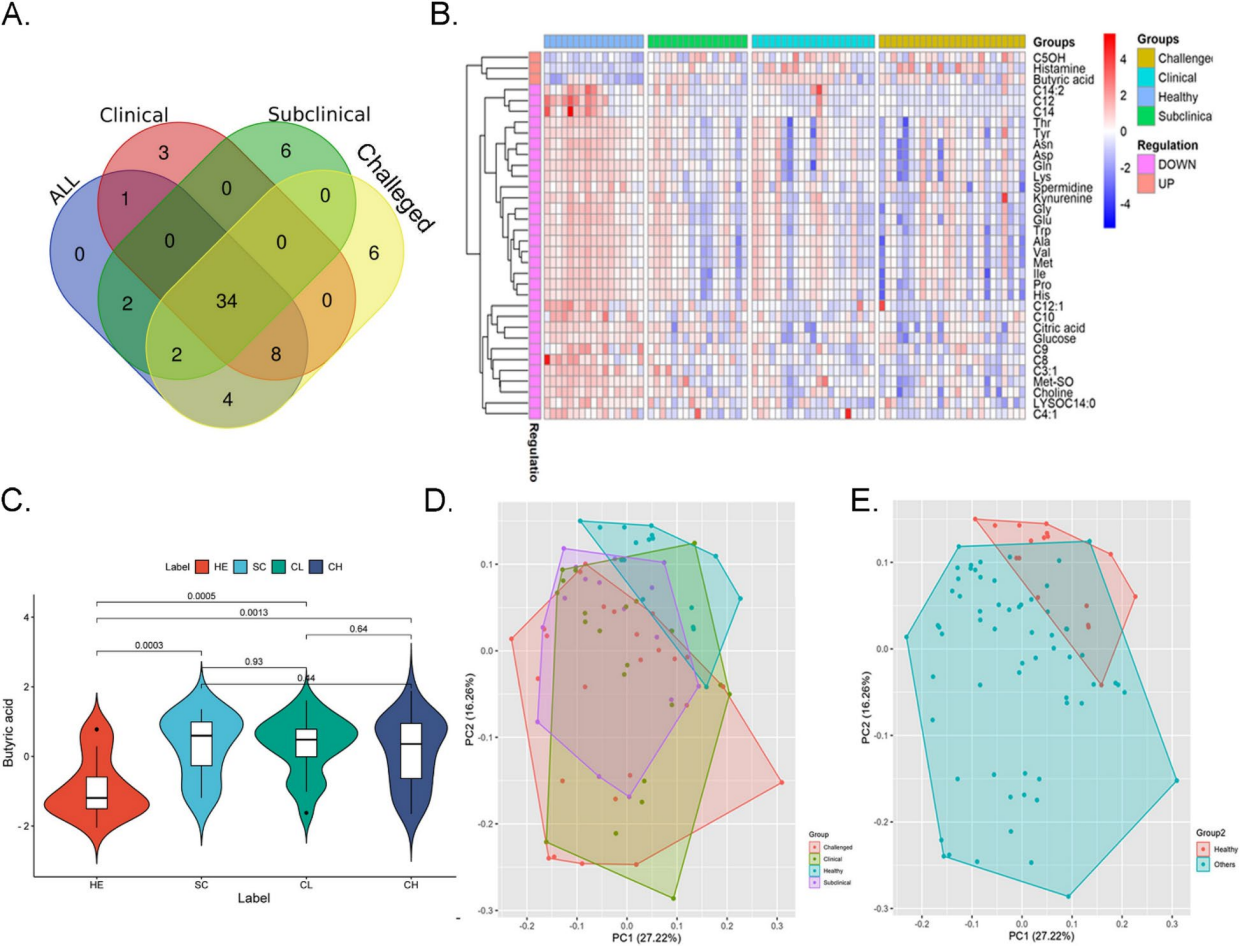
Overview of shared metabolic profiles and metabolic profile alterations in CP-exposed broiler chickens. **A** Venn diagram identifying 34 significant metabolites shared among NE (clinical, subclinical, and CP challenged but no lesions), and control group birds. **B** Heatmap highlighting the differential expression pattern of butyric acid, histamine, and C5OH between NE group versus control birds. **C** Violin plots depict the over expression of butyric acid in CP-challenged NE birds (HE-healthy, SC-Subclinical, CL-Clinical and CH-Challenged) relative to healthy controls (*P* < 0.01 for all comparisons). **D** PCA plots showing variance across different NE groups based on 34 significant metabolites with PC1 accounting for 27.22% and PC2 for 16.26% of the variance. **E** PCA plots showing distinct metabolite profile between NE versus healthy control groups

 Further investigation through a Venn diagram (Fig. 4A) identified 34 metabolites consistently altered across all NE categories (Table S3). Heatmap analysis (Fig. 4B) details the metabolites expression, with upregulation in diseased birds indicated by red and downregulation by green. The dendrogram on the heatmap revealed a pattern of sample clustering, indicating a degree of homogeneity of the metabolites within each NE group.

Among the 34 metabolites analyzed, butyric acid (SCFA) demonstrated robust upregulation (logFC 1.89, *P* < 0.001) across all CP challenged broilers, with its expression intensifying with disease severity. Histamine (a biogenic amine) and the acylcarnitine C5OH, also showed a significant increase in expression, particularly in birds with clinical NE (histamine logFC 2.00, *P* < 0.001; C5OH logFC 0.03, *P* < 0.001).

A significant decrease in long-chain fatty acids (LCFAs) in NE-affected birds was noted in comparison to controls. Minor metabolites C9 and C8 were significantly reduced (logFC −0.02 and −0.03, respectively; *P* < 0.001*)*, in addition to C4:1, C14:2, and C14 (logFC −0.04 to −0.07; *P* < 0.0001). Further, a significant decrease in C10, C3:1, and C12:1, with logFC reaching −0.20 to −0.27 (*P* < 0.0001) was observed, in addition to a significant drop in C12 (logFC −0.31, *P* < 0.0001).

Amino acids, including isoleucine (logFC −0.35, *P* < 0.0001), valine (logFC −1.23, *P* < 0.00001), and tryptophan (logFC −1.37, *P* < 0.0001), exhibited significant reductions, particularly in birds challenged with CP but no lesions. Further, alanine (logFC −0.73, *P* < 0.0001), glycine (logFC −1.30, *P* < 0.0001), histidine, tyrosine, methionine (logFC ranges from −0.76 to −1.42; *P* < 0.0001), proline, glutamate, aspartate (logFC up to −1.75; *P* < 0.0001), threonine, aspartate, and lysine (logFC −1.65 to −1.94; *P* < 0.0001) showed significant reductions across all NE affected birds. In addition, downregulation of glutamine and asparagine (logFC −2.26 and −2.59; *P* < 0.0001) across disease severities.

Energy metabolism metabolites were notably downregulated, including glucose (logFC −2.62, *P* < 0.001) and citric acid (logFC −4.39, *P* < 0.001). In addition, choline was significantly reduced (logFC −1.48, *P* < 0.001), particularly in challenged birds that had no lesions. Compounds integral to cellular health such as spermidine (logFC −1.04, *P* < 0.01), Met-SO (logFC −1.34, *P* < 0.001), and LYSOC14:0 (logFC −0.52, *P* < 0.001) were also decreased across all NE categories.

 A logistic regression model consisting of histamine, butyric acid, citric acid, tyramine, and ornithine showed that butyric acid as the most significant (*P* < 0.001) metabolite with high association with NE infected birds compared to the controls (Table S4). Figure 4C shows the violin plots demonstrate elevated butyric acid levels in the subclinical (*P* < 0.001), clinical (*P* < 0.001), and challenged (*P* < 0.001) groups as compared to healthy controls.

PCA plots help visually elucidate the metabolic distinctions among various groups of chickens. Our PCA plot analysis suggests that metabolic profiling can distinguish between healthy and CP-challenged broiler chickens, as well as between broiler chickens with different severities of NE. In Fig. 4D, a plot of 34 common metabolites shows that PC1 (*x*-axis) explains 27.22% of the variance in the dataset, and PC2 (*y*-axis) explains 16.26%. Together, they provide a combined explanatory power of 43.48% for the dataset variance. The PCA plot demonstrates that the challenged, but lesion-free group exhibits a distinct and consistent metabolic profile separate from healthy control birds, while the overlap between the clinical and subclinical groups indicates a shared metabolic process in NE progression. Figure 4E, the PCA plot illustrates that PC1 and PC2 together capture distinct variations in the metabolic profiles between healthy chickens and those with NE infections indicating specific metabolic shifts along these principal components.

### Jejunal metabolite analysis identifies potential metabolic pathway alterations

**Fig. 5:**
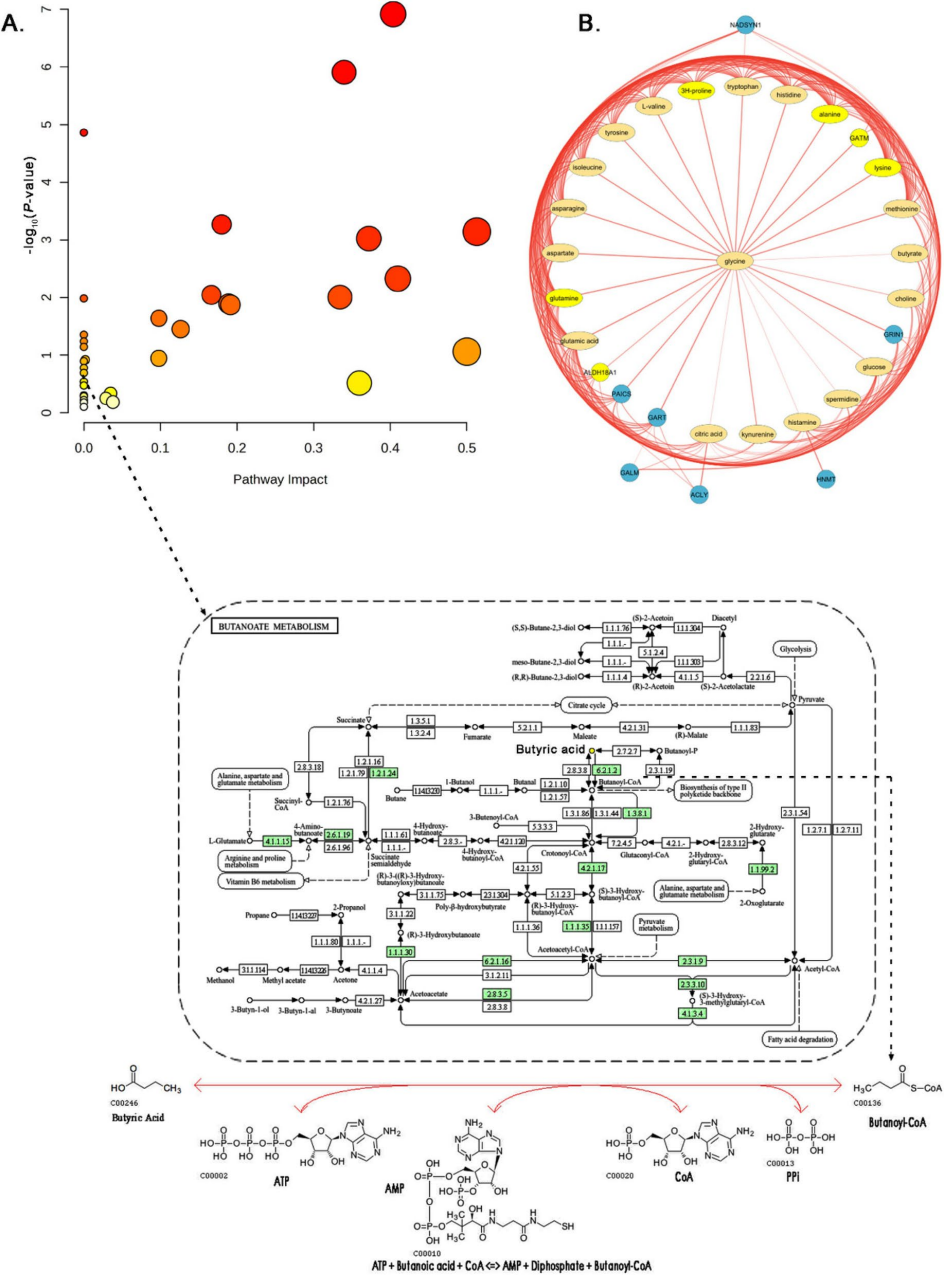
Metabolic pathway analysis and metabolite-protein interaction networks in CP-exposed broiler chickens. **A** Bubble plots of 34 jejunal metabolites within key metabolic pathways, with a focus on the butyric acid metabolism (KEGG pathway: gga00330) in broiler chickens (*P* < 0.05). The size of each bubble corresponds to the significance of the pathway, while its horizontal axis placement reflects the impact score. **B** The metabolite-protein interaction network under CP exposure, using ellipses for metabolites and circles for proteins, illustrating the web of interactions between them. The colour of the bubbles (yellow, orange and red) indicates high, medium and low significance levels across the pathways impacted by the metabolic changes

Pathway analysis conducted on the 34 most significantly altered jejunal metabolites revealed their involvement in multiple metabolic pathways. This was indicated by the ratio of metabolite hits to the total number of metabolites involved in each specific pathway (Fig. 5A). Notably, butyric acid metabolism emerged as one of the most significant pathways affected by the disease (*P* < 0.01). Amino acid metabolism pathways, including aminoacyl-tRNA biosynthesis (*P* < 0.001), arginine biosynthesis (*P* < 0.001), and glycine, serine, and threonine metabolism (*P* < 0.001), were also dysregulated. Energy metabolism pathways such as glyoxylate and dicarboxylate metabolism (*P* < 0.001), glycolysis/gluconeogenesis (*P* < 0.01), and the citrate cycle (*P* < 0.01) were impacted. Additionally, pathways related to the metabolism of key metabolites like glutathione (*P* < 0.001), nitrogen (*P* < 0.001), and tryptophan (*P* < 0.01) were also significantly connected to the metabolites altered in NE birds (Table S5).

### Deciphering jejunal metabolic pathway networks

A detailed network analysis of metabolites and proteins revealed their complex interplay within metabolic pathways providing insights into disease mechanisms at the cellular level. Of the 34 differentially expressed metabolites, we constructed a network encompassing 30 nodes: 21 metabolites (represented as ellipses) and 9 proteins (circles), linked to the central glycine node by 196 interactions (edges) in the STITCH database. The specific metabolites involved include valine (Val), glutamine (Gln), glucose, proline (Pro), glutamate (Glu), tryptophan (glutamate), C8, glycine (Gly), spermidine, kynurenine, threonine (Thr), aspartate (Asp), C10, C14, lysine (Lys), tyrosine (Tyr), citric acid, histamine, choline, alanine (Ala), and methionine (Met). The identified proteins include NADSYN1, GALM, ALDH18A1, PAICS, GART, GRIN1, ACLY, HNMT, and GATM. This network was visualized in radial layout format using the Cytoscape software tool (Fig. 5B). All these interactions had a minimum confidence score of 0.4 as per the data evidence from biological experiments, functional biology databases, and various genomic associations. The enrichment analysis of this network revealed the significance of butanoate (butyric acid), arginine and proline metabolisms (Kyoto Encyclopedia of Genes and Genomes (KEGG) pathway: gga00330), with a *P* < 0.01, as evidenced by the ratio of protein-metabolite hits to the total number of protein-metabolite hits involved in each specific pathway. Of the 9 protein interactors, ALDH18A1 and GATM have shown strong functional enrichment with metabolic pathways involving arginine, proline, and glutamate, which act as precursors to butanoate synthesis via ornithine (Table S6).

### Jejunum metabolite content with NE induced via different CP isolates

No gross or histopathological lesions were observed in the CP unchallenged group (group 1). 58.82% (*n* = 10) of birds in group 2 (*cpa*, *netB*, *cpb2* and *tpeL*) developed subclinical NE, 41.18% (*n* = 7) developed clinical NE and 0% developed no histopathological lesions. In group 3 (*cpa*, *cpb2* and *netB*), 6.25% (*n* = 1) of birds developed subclinical NE, while 6.25% (*n* = 1) developed clinical NE and 87.5% (*n* = 14) had no gross or microscopic NE lesions. In group 4 (*cpa* and *cpb2*), no birds (*n* = 0) developed subclinical NE, while 12.5% (*n* = 2) developed clinical NE and 87.5% (*n* = 14) with no gross or microscopic lesions (Fig. 2E).

**Fig. 6:**
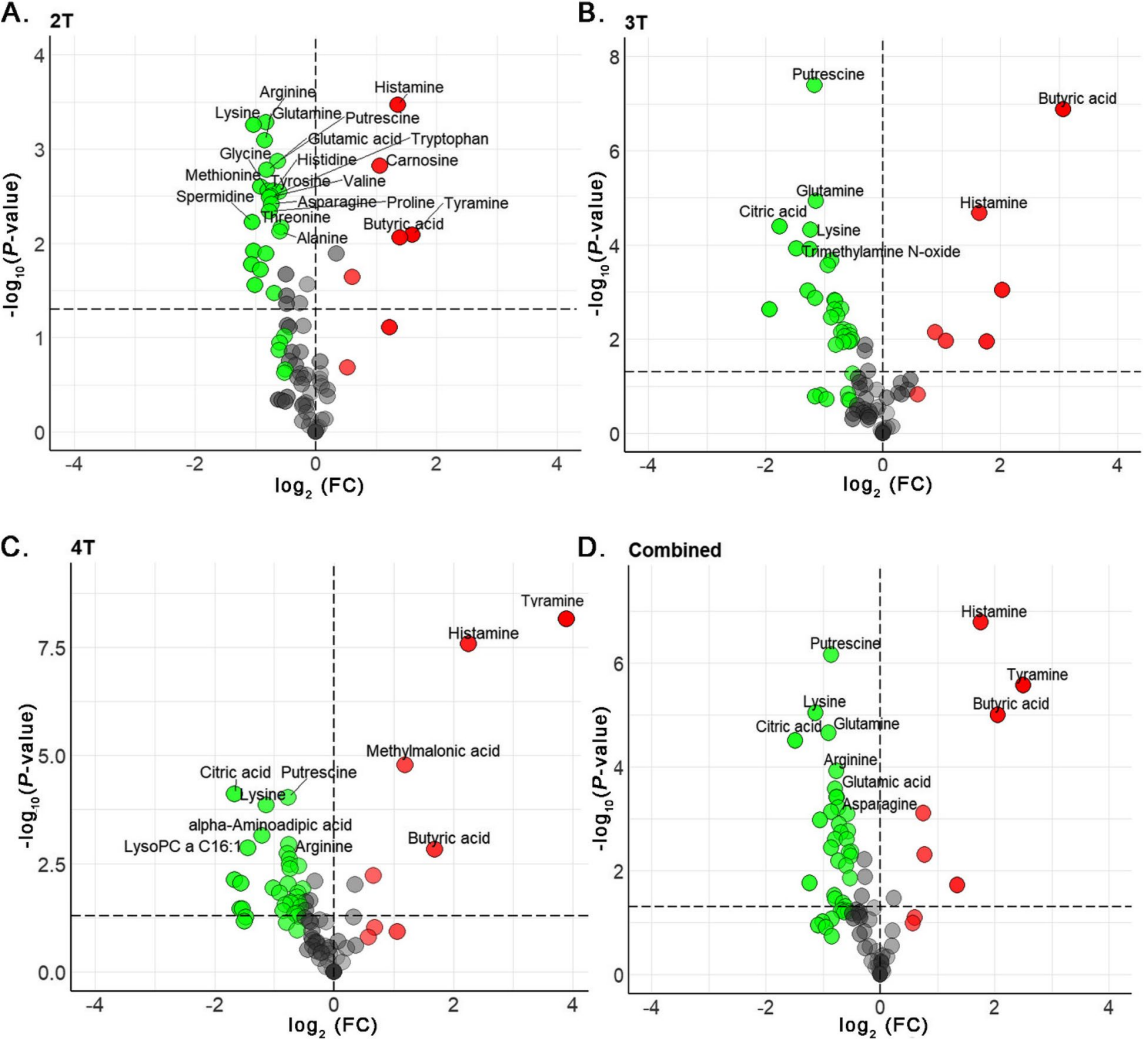
Metabolite expression in broiler chickens challenged with CP with different toxin genes. Volcano Plots (**A**–**D**) illustrate the statistical significance versus fold change of jejunal metabolites across CP isolates with 2T, 3T, 4T and combined toxin gene groups in NE birds compared to controls. Red dots represent significantly upregulated metabolites, and green dots indicate downregulated metabolites, showcasing distinct metabolic profiles associated with NE severity post CP challenge

 The absolute quantities of individual metabolites extracted from NE of birds infected with CP bacterium with different toxin gene combinations, quantified by LC-MS/MS and DFI-MS are detailed in Table S7. Jejunal metabolomic analysis of NE birds revealed a profound reprogramming of metabolic profiles, characterized by a consistent downregulation of several metabolites across CP isolates with 2T, 3T, 4T and combined NE categories compared with healthy controls. This trend was evident in all pairwise comparisons shown in volcano plots, with birds exposed to 2T CP isolate exhibiting 6 upregulated and 27 downregulated metabolites. On the other hand, birds exposed to 3T isolate manifested 6 upregulated and 30 downregulated metabolites, and birds exposed to 4T isolate demonstrated 3 upregulated and 36 downregulated metabolites. Notably, the combined infected group also displayed a similar pattern, with 7 upregulated and 32 downregulated metabolites (Fig. 6A–D).

**Fig. 7:**
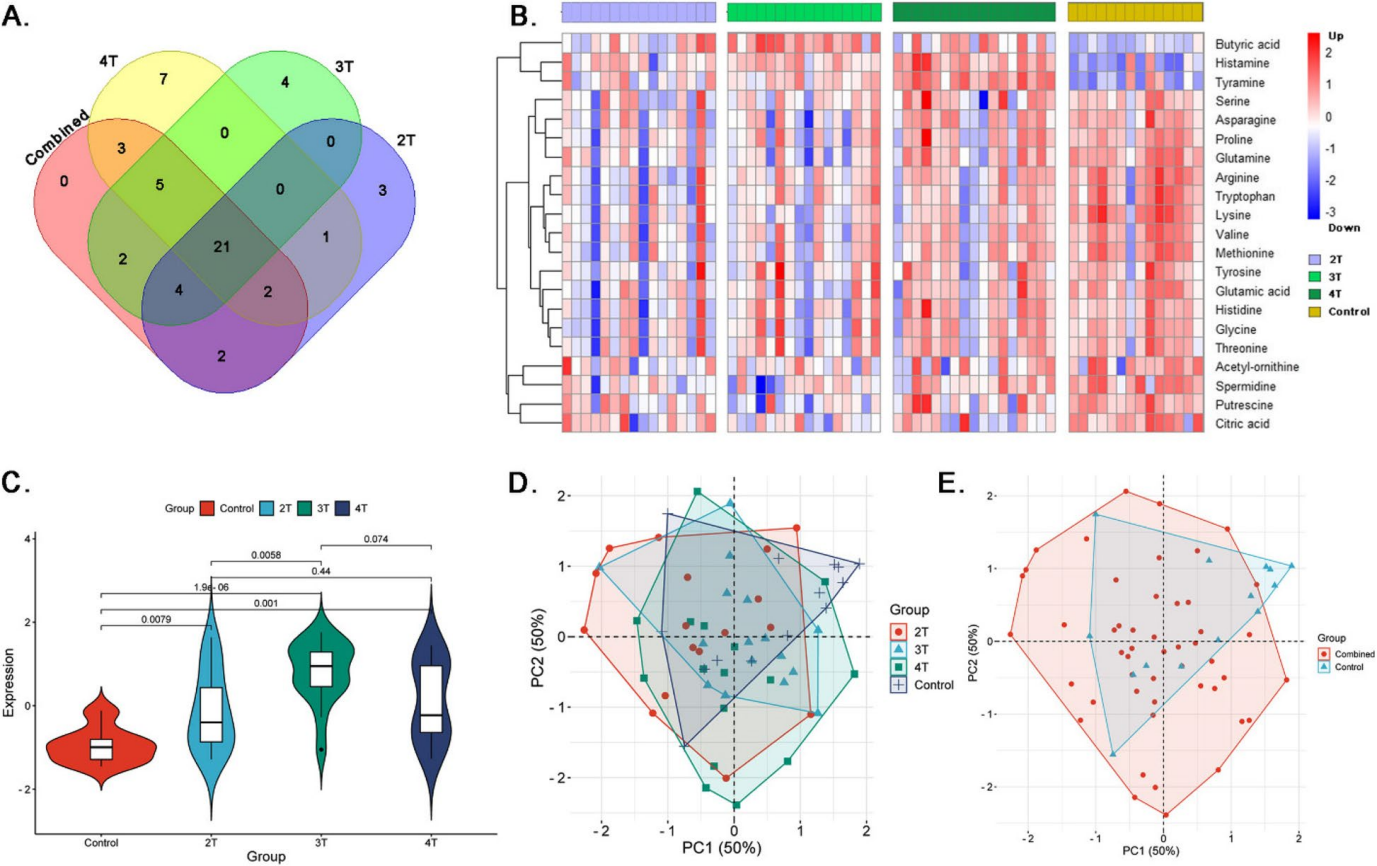
Overview of shared metabolic profiles, their expression patterns, multivariate analysis of metabolites in broiler chickens challenged with CP and its different toxin combinations. **A** Venn diagram identifying 21 metabolites consistently altered across all NE categories. **B** Heatmap showing upregulation of butyric acid, methylmalonic acid and histamine, in clinical, subclinical, and challenged group birds compared to controls. **C** Violin plots depict the over expression of butyric acid in NE birds challenged with different toxin gene numbers (2T, 3T and 4T) relative to healthy controls (*P* < 0.01 for all comparisons). **D** PCA plots showing Variances across NE groups with 21 metabolites; PC1 (50%) and PC2 (50%). Challenged group shows a prominent cluster. **E** Healthy group metabolites are distinctly separated from those of CP infected NE birds, indicating subtle metabolic changes due to infection

 The Venn diagram (Fig. 7A) provides valuable insights into the alteration of 21 metabolites across all CP isolates (2T, 3T, 4T and combined) (Table S8). Among the expression patterns of these 21 metabolites, amines and organic acids exhibit upregulation. Short-chain fatty acids like butyric acid (logFC range: 1.39–3.08), exhibited a marked upregulation across all groups. Interestingly, butyric acid shows its highest expression in response to the 3T challenge. Similarly, histamine (imidazole alkaloid; logFC range: 1.36–2.26) and tyramine (amine; logFC range: 1.60–3.89) display consistent upregulation across all challenged groups.

All amino acids demonstrated downregulation across the 2T, 3T, and 4T toxin gene counts in NE-affected birds, with significant *P* < 0.05 and log fold changes (logFC) below −0.5. Asparagine, glutamine, and lysine displayed an interesting trend between toxin gene count and expression level. These metabolites showed the most substantial downregulation at the 3T challenge group (asparagine logFC −0.94, glutamine logFC −1.14, lysine logFC −1.23), with minimal recovery observed at 4T. In contrast, serine, threonine, and tyrosine exhibited a weaker correlation between toxin gene count and expression level. Their logFC values showed some variation across the challenge groups (serine: −0.49 to −0.57, threonine: −0.56 to −0.65, tyrosine: −0.61 to −0.59), but without a clear trend of increasing or decreasing expression in response to increasing toxin gene burden.

Putrescine and spermidine, essential polyamines, exhibited differential responses. Putrescine showed a strong downregulation (logFC −1.17) at the 3T challenge group. Spermidine’s response might differ (logFC data not provided), possibly exhibiting a weaker correlation or a different downregulation pattern compared to putrescine. Additionally, citric acid, a key player in energy metabolism, displayed substantial downregulation (logFC around −1.76) at 3T, suggesting a possible decrease in energy production in response to the toxin burden. Finally, acetylornithine, a vital intermediate in arginine synthesis, also exhibited downregulation across the challenge groups. The logFC values for acetylornithine likely range from −0.68 to –0.80 across the 2T, 3T, and 4T groups. Lastly, glutamic acid and methionine show a consistent degree of downregulation across all gene counts (glutamic acid logFC around −0.8, methionine logFC −0.91 at 2T to –0.76 at 4T), indicating a possible threshold effect.

The heatmap (Fig. 7B) illustrates distinct variations in the jejunal metabolomes of NE-affected birds, revealing complex responses to CP isolates with various combinations of toxin genes (*cpa*,* cpb2*,* netB*, and *TpeL*). Metabolites such as butyric acid, histamine, and tyramine are overexpressed (shown in red) across all toxin-exposed groups compared to the control.

 Logistic regression analysis employing butyric acid, lysine, tyramine, citric acid, acetyl-ornithine, putrescine, spermidine metabolites has identified the statistically significant positive association with butyric acid, with a coefficient of 1.23 and a *P* < 0.01 highlighting it as contributor to the NE pathology. Although tyramine exhibited a positive coefficient as well, it’s association did not reach statistical significance. On the other hand, lysine, citric acid, acetyl-ornithine, putrescine, and spermidine were associated negatively, suggesting an inverse relationship with the condition, but these findings were not statistically significant **(**Table S9). The violin plot in Fig. 7C reveals a significant increase in butyric acid levels in the birds challenged with CP isolates of different toxin genes 2T (*P* < 0.001), 3T (*P* < 0.001), and 4T (*P* < 0.001) compared to the control group birds.

In Fig. 7D, illustrates the PCA analysis for metabolomic profiling of broiler chickens based on varying CP toxin gene dosages (2T, 3T, and 4T) and control groups. The clustering shows the variation in metabolomic data, with PC1 explaining 50% and PC2 explaining another 50% of the variance. Clusters for each group (2T, 3T, and 4T) seem to partially overlap with each other but are generally distinct from the control group, suggesting a differentiated metabolomic impact due to NE which may not increase linearly with the toxin gene count. The PCA plot in Fig. 7E effectively differentiates the metabolomic profiles of chickens across the treated (combined toxin genes) and control groups, as evidenced by the distinct clustering pattern. Notably, both PC1 and PC2 capture a significant portion of the variance (50% each), indicating a robust representation of the metabolic alterations. This clear separation between the control and CP-treated groups suggests that the presence of CP leads to a distinct metabolic signature regardless of the type of toxin gene combinations.

### Metabolic pathway alterations in NE-affected broilers induced by different CP isolates

**Fig. 8:**
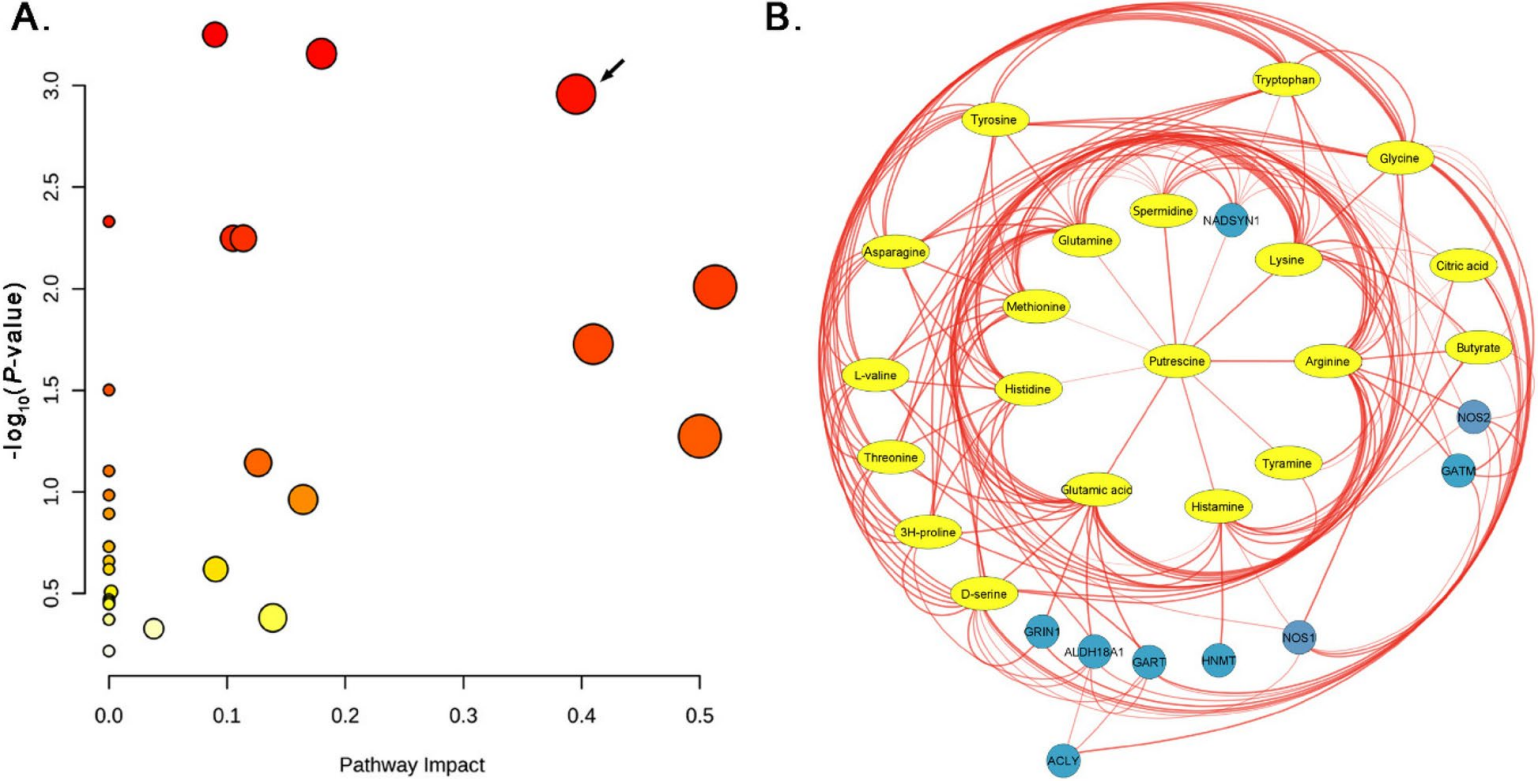
Metabolic pathway analysis and metabolite-protein interaction networks in broiler chickens challenged with CP and its different toxin combinations. **A** Bubble plots of 21 jejunal metabolites within key metabolic pathways, with a focus on the Arginine-Proline metabolism (KEGG pathway: gga00330) in broiler chickens. The size of each bubble corresponds to the significance of the pathway, while its horizontal axis placement reflects the impact score. Arrow mark shows the enrichment of butyric acid pathway. **B** The metabolite-protein interaction network under CP (different toxin gene combinations) exposure, using ellipses for metabolites and circles for proteins, illustrating the web of interactions between them. The radial layout highlights Arginine and Proline metabolite interactor proteins (GATM, ALDH18A1, NOS1 and NOS2) with their first neighbor metabolites (Gln, Pro and Arg)

Notable metabolic shifts were evident in the jejunum of broiler chickens afflicted with NE caused by different CP isolates. Glyoxylate and dicarboxylate metabolism (*P* < 0.001), arginine biosynthesis (*P* < 0.001), and Arginine and proline metabolism (*P* < 0.001) were among the significant pathways (Fig. 8A, and Table S10).

### Network analysis based on metabolite-protein-analysis

A radial network map visualized interactions between 21 jejunal metabolites (ellipses) and 14 associated proteins (circles) (Fig. 8B). Red lines depict functional relationships, with prominent connections observed for histamine, butyrate, lysine, putrescine, and tyramine. The network’s complexity is evident with 81 edges, suggesting a tightly interwoven web of interactions between these molecules. Notably, a minimum confidence score of 0.4 ensures a degree of reliability for the depicted interactions. This network map provides a valuable window into the jejunal metabolic landscape, offering insights into the intricate interplay between metabolites and proteins that govern intestinal function. This radial network analysis highlighted the enrichment in arginine and proline metabolism (KEGG pathway: gga00330) as evidenced by *P* < 0.01. This network also highlighted the interaction between GATM, ALDH18A1, NOS1 and NOS2 proteins and the metabolites like glutamine, proline, and arginine (Table S11).

## Discussion

Our first objective was to explore the jejunal metabolome and metabolic pathways associated with both subclinical and clinical NE following CP challenge with a CP isolate containing toxin genes *cpa*, *netB*, *cpb2* and *TpeL*. Our second objective was to explore the jejunal metabolome and metabolic pathways associated with subclinical and clinical NE following CP challenge with 3 different CP isolates containing *cpa*, *cpb2*,* netB* genes; *cpa*,* cpb2* genes and *cpa*, *netB*, *cpb2* and *TpeL* genes. This experiment was performed to determine if different CP strains could induce the production of different metabolites. This approach was designed to help elucidate whether the metabolomic landscape of NE depends on specific CP isolates (with a different combination of toxin genes or other CP virulence factors). Based on our metabolomics data, it is clear that butyric acid increased in the jejunum of birds with either subclinical (microscopic lesions) or clinical (macroscopic lesions) NE irrespective of the severity of NE. Similarly, butyric acid is elevated in the jejunum of birds with NE lesions caused by CP isolates with any combination of toxin genes (*cpa*, *cpb2*,* netB* genes; *cpa*,* cpb2* genes or *cpa*, *netB*, *cpb2, TpeL* genes). This suggests a role of virulence factors beyond *cpa*, *cpb2*, *netB* and *TpeL* toxins.

CP possesses diverse factors including enzymes (collagenases, hyaluronidases), adhesion molecules, quorum sensing molecules, iron acquisition systems, and regulatory proteins (VirR/VirS) that influence pathogenicity [30–33]. Studies support the role of these factors in toxin production and survival. Understanding these virulence factors and their impact on host metabolism is crucial for developing effective NE control strategies. Furthermore, we have noted that butyric acid is elevated in CP-challenged (with any combination of CP virulence genes) birds with no macroscopic or microscopic NE lesions. Based on this observation, it appears that butyric acid elevation in broiler chickens indicates a vital adaptive mechanism against CP-induced tissue damage in NE, suggesting its potential as an early marker for the disease onset.

In the first experiment, upregulation of butyric acid, histamine, tyramine, and citric acid in CP challenged birds suggested a potential role for these metabolites in NE lesion development. Butyric acid and histamine demonstrated a significant upregulation in all NE infected birds regardless of their clinical severity. Interestingly, butyric acid expression increased from subclinical to clinical NE lesions and can act as a potential biomarker for assessing NE disease progression. Past studies on the pathogenesis of NE in preterm neonates noted that SCFAs are generated by several bacterial species, especially *Clostridium* species [34]. Butyric acid is the main SCFA generated under anaerobic and acidic conditions, preferred by high concentrations of hydrogen. Hence, higher concentrations of butyric acid could directly damage the intestinal mucosa and induce T-cell apoptosis [35, 36], which is in line with the findings of our study. Similarly, butyrate/butyric acid production is due to fermentation of dietary non-digestible carbohydrates by bacteria in the intestines of broiler chickens [37]. Bacterial families containing strict anaerobes such as Ruminococcaceae, Lachnospiraceae and clostridial subphylum *Clostridium* clusters I and XVI induce butyric acid production [38, 39]. In addition, *Butyricicoccus pullicaecorum* has been identified as butyrate producer in the cecal content of 4-week-old broiler chickens [40]. Therefore, the observed increase in butyric acid in the jejunum following CP challenge can be primarily attributed to dysbiosis-induced shifts in gut microbiota favoring butyrate-producing bacteria, which metabolize dietary fibers. Additionally, the high-protein diet employed in the study may promote protein fermentation, further contributing to elevated butyric acid levels. These changes reflect the intricate interplay between diet, microbial ecology, and pathogen activity in the gut. Based on our findings, we hypothesize that butyric acid stimulates intestinal mucin secretion, which is further utilized by bacteria like CP to adhere onto mucosal epithelium and proliferate [41]. CP has the ability to use mucous as an energy source as it contains sialidases; NanI (77 kDa), NanJ (129 kDa) and NanH (43 kDa) [42]. The generation time of CP is less than 10 min [43] which further contributes to the massive release of toxins in a short span of time. These events overcome the favorable effects of butyric acid on the tight junctions of intestinal epithelial cells. In addition, increased permeability facilitates the translocation of bacterial toxins into the bloodstream, exacerbating NE in broiler chickens. In poultry, butyric acid has been utilized as a feed supplement for its beneficial effects in maintaining the intestinal barrier function via the up-regulation of tight junction protein claudin-1 transcription [44]. NE infection disrupts the intestinal barrier function by downregulating the claudin-4, *ZO-1*, occludin, *LEAP-2* and mucin-2 gene expression in the jejunum. However, it has been shown that supplementation with dietary microencapsulated sodium butyrate stimulated the tight junction proteins and antimicrobial peptide *LEAP-2* gene expression [37]. Butyric acid also has the capability to reduce the expression of cytokine genes such as *IL1β*, *IL2*, *IL6*, *IL8*, and *IL12* as well as tumor necrosis factor α (*TNFα*), by inhibiting prototypical proinflammatory signaling nuclear factor NF-κB pathway [45]. However, overproduction of butyric acid can deteriorate the gut health [2]. Moreover, the synergy between over production of butyric acid and CP infection appears to exacerbate damage to the intestinal epithelium, creating an environment conducive to intestinal necrosis and disease progression. Further, higher dietary levels of organic acid blends of calcium butyrate, fumaric acid, citric acid and medium-chain fatty acids have shown to reduce the productive performance of broiler chickens leading to higher mortality, culling rates, and increased CP shedding in feces post CP challenge [46].

Interestingly, butyric acid and acetic acid have shown to be associated with decreased growth and both increased colon wet weight and histologic lesion scores, in newborn rats [47]. The pathology seen in rats was similar to neonatal necrotizing enterocolitis. Hence, overproduction of SCFAs might have a role in the pathogenesis of necrotizing enterocolitis in premature infants [47]. In addition, premature infants have insufficient lactase enzymes and increased SFCA production due to carbohydrate malabsorption, microbial overgrowth further lowering the pH of the intestine. Lower pH led to proliferation of *Clostridium* and acts as a predisposing factor for NE [48]. In one trial, butyric acid, when combined with medium-chain fatty acids and essential oils, reduced severity of gross NE lesions. However, higher concentrations of these metabolites was ineffective against NE [49].

Histamine is a biogenic amine and acts as a mediator of allergic as well as non-allergic inflammatory processes in the gut mucosa. Similar to butyric acid in the gut, histamine can play a protective or negative role against bacterial infections [50]. In higher concentrations, histamine contributes to mucosal inflammatory disorders such as irritable bowel syndrome, inflammatory bowel disease and histamine intolerance [51]. The microbiome is also a potential source of histamine production along with immune cells such as mast cells or basophils. A previous study reported significantly higher abundance of histamine-secreting bacteria like *Clostridium perfringens*, *Enterococcus faecalis*, genus *Staphylococcus*, *Proteus*, and unidentified genera of *Enterobacteriaceae* in histamine intolerant patients as compared to healthy individuals [52]. Similarly, our findings suggest that CP proliferation and the associated microbiome imbalance potentially induced an increase in histamine levels in the jejunum of NE affected birds.

Interestingly, amino acids such as isoleucine and valine were significantly (*P* < 0.0001) reduced in birds with no NE lesions after CP challenge. This indicates an early metabolic alteration before the appearance of microscopic disease. These branched-chain amino acids support the proliferation of pathogenic bacteria through protein synthesis. It is well known that CP lacks amino acid synthesis machinery, hence proliferation and colonization requires amino acid availability [53]. In NE infected birds, both amino acid and fatty acid metabolism was downregulated which can be due to utilization by CP during gut colonization. The doubling time of CP is less than 10 min hence, nutrient demand is significantly elevated. Also, amino acids and fatty acids are integral parts of Gram-positive cell membranes and cell wall respectively [54]. Choline, a biomarker of cell membrane turnover and nucleotide metabolism, was significantly reduced particularly in birds challenged with CP but had no lesions, suggesting potential membrane integrity and signaling pathway dysregulation [55].

Energy metabolism metabolites were notably reduced, with glucose (*P* < 0.001) and citric acid (*P* < 0.00001) indicating energy deficits and impaired citric acid cycle function. In contrast, glycogen-synthesis-pathway and acetyl-CoA pathways were shown to be elevated in cecal transcriptome analysis of birds co-infected with CP and *Eimeria* oocysts [56]. Conversion of butyric acid to acetyl-CoA via β-oxidation in the mitochondrial matrix could compensate for the partial energy requirements [46] within the gut of CP-infected birds. Such metabolic adaptations are critical in understanding the pathogenic mechanisms of CP, as they link directly to both tight junction integrity and energy metabolism pathways. Butyric acid interaction with the tight junction proteins suggests its influence on intestinal barrier integrity [57]. These findings underscore the significant role of butyric acid in the pathogenesis of NE with elevated levels correlating with NE severity.

In our second experiment, despite the challenge of birds with different CP isolates with different toxin gene combinations, the metabolic profile remained similar regardless of the isolate used for challenge. Notably some metabolite changes, like butyric acid and citric acid, were consistent across disease severities, while other metabolites, including methylmalonic acid showing increasing upregulation with fold changes ranging from 0.60 to 1.04. These metabolomic findings highlight disruptions in fundamental metabolic processes such as protein metabolism, amino acid metabolism, and antioxidant defenses in CP infected birds. Additionally, the enrichment of arginine biosynthesis signifies its importance in nitrogen metabolism and immune function indicating perturbations in essential cellular pathways and immune responses amidst NE development.

**Fig. 9:**
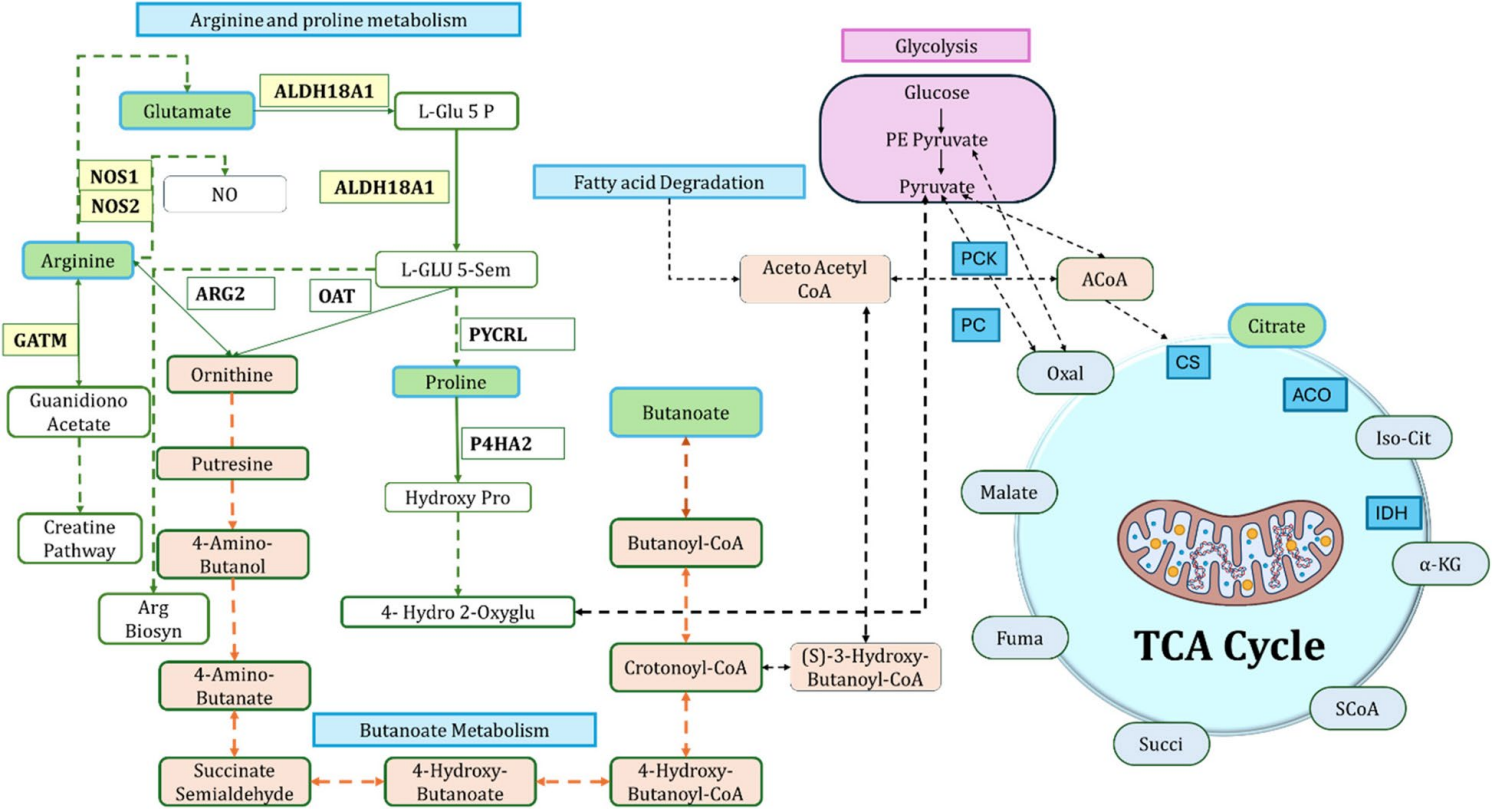
Integrated metabolic pathway network analysis in NE-affected jejunal tissues. This schematic represents the intricate metabolite-protein networks highlight the involvement of metabolites [Arginine, Glutamate, Proline, Butyric Acid, and Citrate (in green color)] and proteins (GATM and ALDH18A1 shown in yellow and PC, PCK, CS, ACO and IDH shown in turquoise). Glutamate acts as a precursor to arginine, proline, and butanoate metabolites. Butanoate is further connected to acetyl Co-A, indicating metabolic adaptations in pathophysiology of CP induced NE in broiler chickens

Protein-metabolite network analysis can be used to improve the identification of disease-associated metabolic pathways beyond metabolite-centric approaches. Network analysis of metabolites in CP-induced NE chickens highlighted interactions between GATM, ALDH18A1, NOS1 and NOS2 proteins. These are enriched in arginine-proline and glutamate metabolism, potentially providing precursors to butyrate synthesis by butanoate metabolism (Fig. 9) [58]. This is significant because proline, which is derived from glutamate, fuels protein synthesis, polyamine production, and the nitric oxide pathway. All of these are crucial for intestinal health and mediating inflammatory responses [59]. The observed increase in butyrate could be a consequence of either direct CP-mediated fermentation or a shift in gut microbiota favoring butyrate producers, potentially aimed at restoring gut health and repairing tissue damage [60]. Interestingly, the increased butyric acid levels and enrichment of arginine-proline metabolism observed in jejunum exposed to CP isolates of different toxin genes points us to postulates the connectivity between arginine-proline and the TCA metabolisms via ornithine and butanoate. The TCA cycle is a key pathway for cellular energy production, utilizes acetyl-CoA derived from various sources. The reduced citrate metabolite levels as seen in our data suggests potential CP toxin effects or cellular damage that limits substrate availability [61]. Furthermore, CP infection might promote a hypoxic environment favoring CP while stressing oxygen-dependent gut bacteria. Reduced citrate levels could indicate a shift away from aerobic energy metabolism, potentially due to tissue damage or inflammation [62]. Overall, our protein-metabolite network analysis revealed a metabolic adaptation of jejunal tissues in CP infection, disrupted TCA cycle function, and a potential compensatory mechanism involving increased butyrate production to maintain energy homeostasis in compromised jejunal tissues.

## Conclusion

Our study indicates that jejunal butyric acid levels rise significantly in broilers with NE, suggesting its potential as a sensitive predictor of disease occurrence. This elevation occurs irrespective of NE severity or CP toxin gene profiles. Notably, under our experimental conditions, there was no increase in butyric acid levels in the control group, emphasizing the specificity of this response in NE-affected birds. Metagenomics analysis revealed that NE-affected birds exhibited up to 70%–90% CP colonization, highlighting a substantial source of butyric acid. Butyric acid’s elevation could also be affected by dietary influences, such as high-fiber intake or probiotics, further validation studies under field conditions are essential to ascertain its diagnostic utility. Additionally, examining other NE triggers (like coccidia or mycotoxin pre-infection) could expand our understanding our understanding of the disease’s etiology. Although butyric acid shows promise as an NE indicator, validation under field conditions should integrate clinical symptoms and other laboratory tests to ensure a comprehensive evaluation.

## Supplementary Information

**Supplementary Material 1: Table S1** Composition of feed used in the study. **Table S2** Metabolite ratios determined across different NE birds. **Table S3** 34 differentially expressed metabolites across different NE categories of birds versus healthy control birds. **Table S4** Logistic regression analysis of key metabolites identified in *Clostridium perfringens* infected birds. **Table S5** Pathway analysis of 34 metabolites identified in NE birds. **Table S6** Metabolite-protein interaction network analysis of 34 jejunal metabolites exposed to CP infection. **Table S7** Metabolite ratios determined across different NE birds challenged with CP with different toxin gene numbers. **Table S8** Comparative analysis of 21 metabolites in broiler chickens infected with CP isolates with different gene combinations (2T, 3T, 4T and combined). **Table S9** Logistic regression analysis of key metabolites identified in *Clostridium perfringens* infected birds. **Table S10** Pathway analysis of 21 jejunal metabolites identified in NE birds treated with CP isolates with toxin genes combinations. **Table S11** Metabolite-protein interaction network analysis of 21 jejunal metabolites exposed to CP infection with different toxin gene combinations.

## Data Availability

The data supporting the findings of this study are available from the authors upon request.

## Abbreviations

3-NPH: 3-Nitrophenylhydrazine
3T: 3 toxin genes
4T: 4 toxin genes
ACU: Animal Care Unit
Ala: Alanine
Asp: Aspartate
BHT: Butylated hydroxytoluene
COX: Cyclooxygenase
CP: *Clostridium perfringens*
CPA: CP alpha toxin
CPB: CP beta toxin
CPE: CP enterotoxin
DFI-MS: Direct flow injection mass spectrometry
DI: Direct injection
ETX: Epsilon toxin
FDR: False discovery rate
Gln: Glutamine
Glu: Glutamate
Gly: Glycine
IL: Interleukin
ISTD: Internal standard mixture
ITX: Iota toxin gene
KEGG: Kyoto Encyclopedia of Genes and Genomes
LCFAs: Long-chain fatty acids
LC-MS: Liquid chromatography-mass spectrometry
LOD: Limit of detection
Lys: Lysine
Met: Methionine
MRM: Multiple reaction monitoring
NAD: Nicotinamide adenine dinucleotide
NE: Necrotic enteritis
NetB: Necrotic enteritis B like toxin
PCA: Principal component analysis
PITC: Phenyl isothiocyanate
Pro: Proline
RWA: Raised without antibiotics
SCFAs: Short-chain fatty acids
Th2: T-helper 2
Thr: Threonine
TMIC: The Metabolomics Innovation Centre
TNFα: Tumor necrosis factor α
TpeL: Large clostridial toxin
Trp: Tryptophan
Tyr: Tyrosine
UHPLC: Ultra-high-performance liquid chromatography
Val: Valine
WCVM: Western College of Veterinary Medicine
